# Exploration of alpha-glucosidase inhibitors: A comprehensive in silico approach targeting a large set of triazole derivatives

**DOI:** 10.1371/journal.pone.0308308

**Published:** 2024-09-06

**Authors:** Oussama Abchir, Meriem Khedraoui, Imane Yamari, Hassan Nour, Abdelkbir Errougui, Abdelouahid Samadi, Samir Chtita

**Affiliations:** 1 Laboratory of Analytical and Molecular Chemistry, Chemistry, Research, and Development, Sciences and Applications, Faculty of Sciences Ben M’Sik, Hassan II University of Casablanca, Sidi Othman, Casablanca, Morocco; 2 Department of Chemistry, College of Science, United Arab Emirates University, Al Ain, United Arab Emirates; University of Education, PAKISTAN

## Abstract

**Background:**

The increasing prevalence of diabetes and the side effects associated with current medications necessitate the development of novel candidate drugs targeting alpha-glucosidase as a potential treatment option.

**Methods:**

This study employed computer-aided drug design techniques to identify potential alpha-glucosidase inhibitors from the PubChem database. Molecular docking was used to evaluate 81,197 compounds, narrowing the set for further analysis and providing insights into ligand-target interactions. An ADMET study assessed the pharmacokinetic properties of these compounds, including absorption, distribution, metabolism, excretion, and toxicity. Molecular dynamics simulations validated the docking results.

**Results:**

9 compounds were identified as potential candidate drugs based on their ability to form stable complexes with alpha-glucosidase and their favorable pharmacokinetic profiles, three of these compounds were subjected to the molecular dynamics, which showed stability throughout the entire 100 ns simulation.

**Conclusion:**

These findings suggest promising new alpha-glucosidase inhibitors for diabetes treatment. Further validation through in vitro and in vivo studies is recommended to confirm their efficacy and safety.

## Introduction

The incidence of diabetes mellitus, commonly known as diabetes, has been increasing, where 537 million people worldwide are living with Diabetes mellitus at 2021 [1], highlighting the need for more effective treatments [2]. The second type is the most common form, accounting for 90% of all cases, as reported by the International Diabetes Federation.

Factors such as obesity, inactivity, and poor diet contribute to the development of type 2 diabetes [3]. Further, the diabetes symptoms can vary depending on the type and individual circumstances, but common ones include frequent urination, excessive thirst, unexplained weight loss, fatigue, blurred vision, and numbness or tingling in the hands and feet [4]. If left untreated or poorly managed, diabetes can lead to various complications, including cardiovascular diseases, kidney damage, nerve damage (diabetic neuropathy), eye problems (diabetic retinopathy), foot complications, and an increased risk of other health issues such as skin infections, hearing impairment, and mental health conditions [5, 6].

Among the current strategies for treating diabetes, the inhibition of alpha-glucosidase is a key approach [7]. Alpha-glucosidase, an enzyme found in the brush border of the small intestine, catalyzes the final step in carbohydrate digestion [8]. It hydrolyzes oligosaccharides and disaccharides into monosaccharides, which are then absorbed into the bloodstream [9]. Inhibiting alpha-glucosidase delays the breakdown and absorption of carbohydrates, leading to a slower and reduced increase in postprandial blood glucose levels [10].

Although Acarbose and Voglibose are commonly used drugs for type 2 diabetes treatment due to their ability to inhibit alpha-amylase and alpha-glucosidase enzymes, they have the drawback of causing numerous side effects such as gastrointestinal discomfort and diarrhea [11, 12]. This necessitates the search for alternative medications that can effectively inhibit alpha-glucosidase activity while minimizing undesirable side effects.

Previous studies have shown that triazole derivatives, such as Sitagliptin, Voriconazole, and Fluconazole, have potential as diabetes drugs by inhibiting the enzyme dipeptidyl peptidase-4 (DPP-4), which indirectly reduces glucose levels by stimulating insulin secretion and inhibiting glucagon secretion [13, 14].

Triazoles are five-membered ring compounds with two carbon atoms and three nitrogen atoms expressed by the chemical formula C_2_H_3_N_3_, characterized by alternating π-bonds. They possess structural properties such as moderate dipole character, hydrogen bonding capabilities, ion-dipole, π-π stacking, cation-π, hydrophobic effect, van der Waal forces, rigidity, and stability, making them pharmacologically active substances [15, 16].

Drug repurposing, bioactivity prediction, and virtual screening play crucial roles in speeding up drug discovery by efficiently identifying potential candidates in a cost-effective and time-efficient manner [17–19]. Techniques like molecular docking and machine learning in virtual screening quickly evaluate large chemical libraries against specific targets [20–22], making them essential tools in modern drug development [23, 24]. However, these computational methods are limited by simplified models that may not fully capture biological complexities [25]. Therefore, it’s crucial to validate findings through rigorous in vitro and in vivo studies. These experiments validate the biological activity, selectivity, and safety of promising drug candidates while refining computational models for enhanced accuracy and reliability [26]. In this study, computational methods were employed to forecast the binding affinity and activity of triazole derivatives against the target protein, along with their potential as oral medications [27–29].

## Materials and methods

### Database collection

Based on previous studies [30], triazole derivatives have shown potency in inhibiting the alpha-glucosidase enzyme, which is beneficial in treating diabetes mellitus disease [31]. These findings suggest that compounds with triazole derivatives could be potential candidates for developing medications against diabetes disease. To identify and study such compounds, the PubChem database can be a valuable resource. it provides information on chemical structures and bioactivity of various compounds [32].

Many triazole compounds have been obtained from the PubChem database for further studies, including molecular docking, ADMET, and molecular dynamics simulation. These techniques help analyze the compounds’ characteristics, behavior, and interactions.

### Ligands selection and preparation

To ensure the compounds analyzed in the study meet certain criteria, a primary prefiltering step was performed using Lipinski rules. This step eliminated compounds with at least one violation, resulting in a reduced number of compounds [33]. The retained compounds had a molecular weight of less than 500 Da, a polar surface area of less than 140Å, a partition coefficient of less than 5, and no more than 10 rotatable bonds [34].

To create a collection of compounds, the LigPrep tool in the Maestro program was used. Specific parameters of drug-likeness were applied to optimize the compounds’ structures and improve their conformations. This optimization process enhances the compounds’ characteristics and behavior [35].

### Protein preparation and active site detection

**Fig 1:**
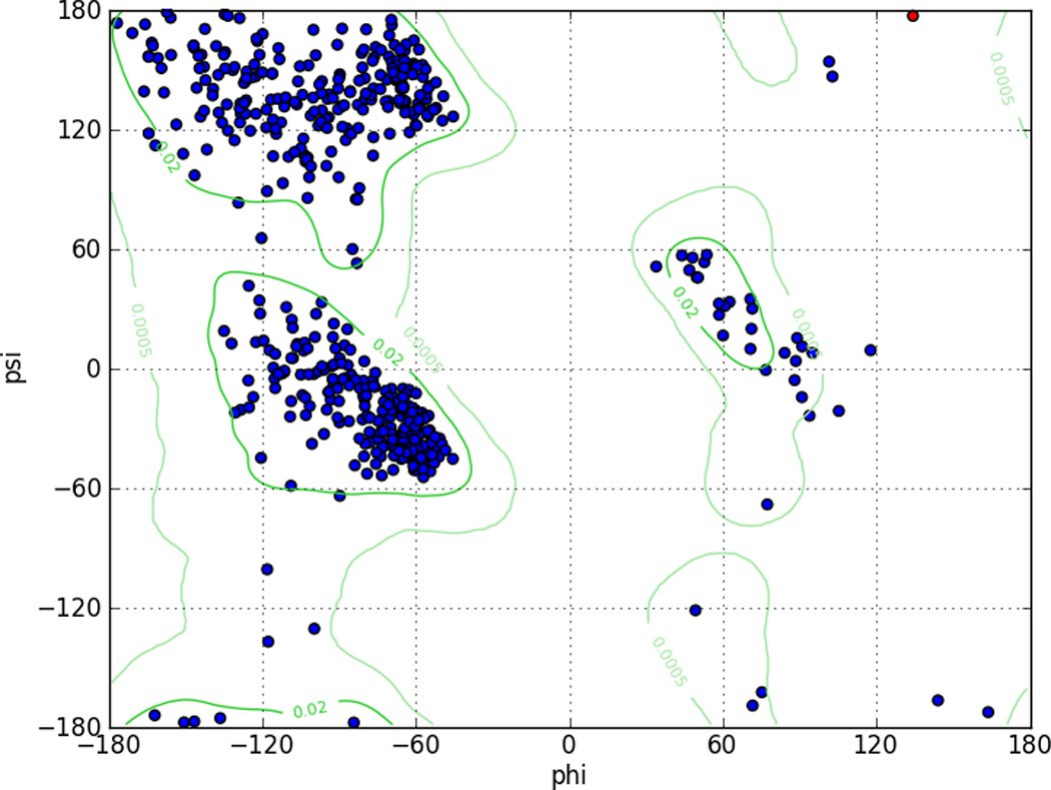
Ramachandran plot of protein structure (3A4A).

The alpha-glucosidase enzyme’s crystal structure, identified as PDB ID: 3A4A and derived from Saccharomyces cerevisiae, was obtained from the Protein Data Bank (PDB) for molecular docking simulations [36]. The Ramachandran plot was used to validate the protein structure to ensure its accuracy before proceeding with further computational analyses. By confirming that the majority of the residues fall within the allowed regions of the plot, we can be confident that the protein model is reliable and that its interactions with potential drug compounds are accurately represented. Other parameters were also assessed including R-free value, Clash-score, Sidechain outliers, and RSRZ outliers which are all considered to be better if they are near to 1 [37]. The Ramchandran plot of the protein structure was obtained by using Chimera software [38]. **Fig 1** illustrates that the majority of residues occupy favorable regions within the Ramachandran plot. This distribution suggests that the protein structure maintains stable conformational characteristics, crucial for its biological function.

**Fig 2:**
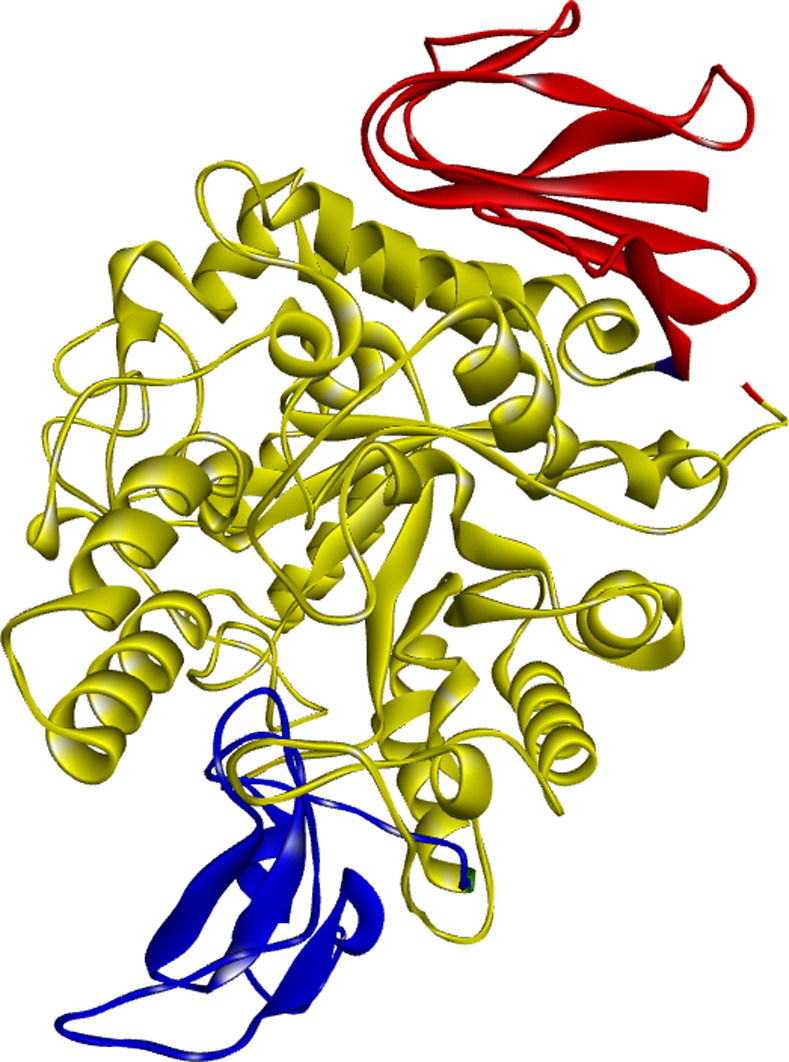
The 3d structure of the analyzed target related to alpha-glucosidase enzyme: Domain A (residues 1–113 and 190–512) represented in yellow, Domain B (residues 114–189) represented in blue, and Domain C (residues 513–589) represented in red.

This particular structure was chosen due to its high resolution of 1.6 Å. The structure of the receptor (3A4A) contains alpha-D-glucopyranose and a calcium ion, which will be removed [39]. The Alpha-glucosidase consists of three domains as presented in **Fig 2**: Domain A (residues 1–113 and 190–512) represented in yellow, Domain B (residues 114–189) represented in blue, and Domain C (residues 513–589) represented in red.

The protein structure underwent refinement using Schrödinger Maestro [40], which involved correcting structural irregularities, optimizing hydrogen bonding, and removing artifacts or non-standard residues to ensure a reliable protein model [41].

To prepare the protein structure for molecular docking simulations, charges and bond orders were assigned, water and solvent molecules were removed, and hydrogen atoms were added to restore correct bonding. Hydrogen bond assignments within the protein structure were optimized to ensure proper formation of critical interactions with potential ligands. The protein’s amino acids were minimized using the OPLS3e force field to optimize the protein’s conformation and stability [42].

The dimensions of the receptor grid box used for molecular docking simulations were determined by analyzing the binding modes of known ligands within the protein’s binding pocket [43]. The receptor grid box size and location were adjusted to concentrate on the desired area of the protein, improving the accuracy and efficiency of the docking process. This step provides information about possible binding mechanisms and guides the creation of novel molecules with improved bioactivity [44].

Using the following coordinates (x = 21.52 Å, y = -7.7 Å, and z = 23.55 Å), a receptor grid was constructed for the prepared protein and placed at the center of the target protein’s binding pocket. The grid box was sized to 20Å in x, y, and z dimensions to provide sufficient space for ligands to be manipulated with various orientations inside the binding pocket.

### Molecular docking, and ADMET analysis

After the initial filtering using Lipinski rules, the molecules were further screened using the GLIDE docking program which consists of three sequential steps: High Throughput Virtual Screening (HTVS), standard precision (SP), and extra-precision (XP). The selection of hit molecules was based on criteria such as docking score, binding energy value, and ligand molecular interactions [45]. These criteria help assess the potential binding affinity and interactions of the molecules with the target [46].

In the field of drug design and development, the pharmacokinetic characteristics of chemical compounds play a vital role. To predict the drug-like properties of selected compounds, the QikProp module within the Schrödinger suite was employed [47]. This module takes into account key pharmacokinetic parameters, including the total solvent-accessible volume measured in cubic Ångströms using a 1.4Å radius probe (ranging from 500.0 to 2000.0), the predicted octanol/water partition coefficient (QPlogPo/w) within the range of -2.0 to 6.5, the apparent Caco-2 cell permeability (QPPCaco) with values below 25 indicating poor permeability and values above 500 indicating excellent permeability, the predicted brain/blood partition coefficient (QPlogBB) within the range of -3.0 to 1.2, the predicted skin permeability (QPlogKp) within the range of -8.0 to -1.0, the number of likely metabolic reactions (#metabol) ranging from 1 to 8, and the predicted percentage of human oral absorption (% Human Oral Absorption) [48, 49]. While other pharmacokinetic parameters were assessed using the Osiris Property Explorer software and SwissAdme-tox online tools [50].

### Molecular dynamics (MD) simulations

MD simulations were performed to study hit compounds identified based on ADMET analysis and molecular docking results. It provides insights into the stability, flexibility, and interactions of molecules over time [51]. Key parameters such as Root Mean Squared Deviation (RMSD), Root Mean Squared Fluctuation (RMSF), and ligand-protein contact were assessed to understand the structural integrity, reliability of binding modes, and overall behavior of the complex [52]. To perform MD simulations, the generated complexes are typically prepared, minimized, and optimized using the OPLS3e force field. the protein preparation wizard provided by the Desmond package in the Schrödinger 2020–3 academic software was used for these purposes.

The simulation setup involves creating an orthorhombic simulation system using the TIP3P water model [53]. To neutralize the charge of the solvated systems, Na+ and Cl- counterions are added. The physiological salt concentration is adjusted to 0.15 M. The system is then gradually heated using the Martina-Tobias-Klein method and the Nose-Hoover thermal algorithm until it reaches the desired temperature, which is set to be below 300 K [54]. The pressure is also controlled and maintained at 1 bar using the isothermal-isobaric ensemble (NPT). Finally, the MD simulation is run for a duration of 100 ns.

## Results

### Lipinski pre-filter

The study involved the analysis of approximately 81,197 compounds retrieved from the PubChem database. These compounds were selected based on their adherence to the Lipinski’s Rule of Five which is widely used in drug discovery to assess the drug-likeness and potential for oral medication of compounds. It states that a compound should not violate at least one of the Lipinski rules. The application of these rules as an initial filter helps to identify compounds with potential for use as oral medications. By adhering to these guidelines, compounds are more likely to possess favorable pharmacokinetic properties, which can contribute to their efficacy and safety.

### Molecular docking

In order to minimize the size of the analyzed compounds and ensure reliable results, a molecular docking approach was employed. The docking process consisted of three sequential steps: High Throughput Virtual Screening, Standard Precision, and Extra Precision docking. The reliability of the obtained results was ensured by applying a multi-step docking approach, starting with a fast-screening step and gradually refining the binding poses and scoring. This approach increases the accuracy of the predicted binding interactions and helps in identifying promising compounds for further experimental validation.

During the HTVS docking step, the compounds were screened using a fast-docking algorithm to identify potential binding poses. The top 10 percent of the best docked ligands based on HTVS scoring were selected for further analysis.

In the SP docking step, a more exhaustive sampling of the ligand conformations was performed. The selected ligands from the HTVS step were subjected to SP docking to obtain more accurate binding poses and scoring.

Finally, the XP docking step was carried out on the top 10 percent of the best docked ligands from the SP docking. XP docking employed an anchor-and-grow sampling approach and a different scoring function to further refine the binding poses and obtain the most accurate results.

By following this protocol, the size of the compound database was significantly reduced from 81,197 to 69 compounds. This reduction in size allowed for a more focused analysis of the compounds with the highest potential for further studies, such as molecular dynamics simulations and ADMET analysis**. Table 1** presents a selection of 69 compounds with good binding affinity for a range of -10.55 to -8.85 kcal/mol, and good glide energies which are less than -40.00 kcal/mol. The compounds are listed along with their SMILES formats, their binding affinity, and their glide energies given insight into their potential effectiveness in interacting with macromolecular targets.

**Table 1:** The docking scores of docked compounds in the binding site of target.

N	Molecular formula	SMILES	Glide energy (Kcal/Mol)	Docking Score (Kcal/Mol)
C1	C_21_H_33_N_7_O	CC1 = CC (= CC (= C1)N2CCN(CC2)C(C)C)NC (= O)C3 = CN(N = N3)CCCCN	-84.37	-10.55
C2	C_20_H_23_FN_6_O_3_	COC (= O)C(CC1 = CNC2 = C1C = CC (= C2)F)NC (= O)C3 = CN(N = N3)CC4CCCN4	-89.32	-10.42
C3	C_19_H_26_FN_7_O	CN1CCN(CC1)C2 = CC (= CC (= C2)CNC (= O)C3 = CN(N = N3)CC4CNC4)F	-92.33	-10.20
C4	C_19_H_26_N_6_OS	C1CC(CNC1)NS (= O)C2 = NC (= NN2)NC3 = C4CCCC4 = CC5 = C3CCC5	-94.98	-10.13
C5	C_17_H_18_F_4_N_6_O	CC1(CC(C(N = C1N)(C)C2 = C(C = CC (= C2)CN3C = NC (= N3)C (= O)N)F)(F)F)F	-61.33	-10.12
C6	C_15_H_25_N_5_O_2_	CCOC (= O)C1 = NNC (= N1)C2CCN(CC2)C3CCNCC3	-75.07	-10.09
C7	C_15_H_21_N_5_O_2_	CC(CCC1 = CC = C(C = C1)O)NC (= O)C2 = CN(N = N2)CCN	-75.27	-10.08
C8	C_3_H_3_N_5_	C1 = NC (= NC = N)N = N1	-42.45	-10.08
C9	C_19_H_25_N_7_O_2_	CC1 = CC2 = C(C (= C1C)NC (= O)C3 = CN(N = N3)CCC4CCCCN4)NC (= O)N2	-86.92	-10.04
C10	C_24_H_29_N_7_O	CC1CCN(C1)CC2 = CC = C(C = C2)CN3C = C(N = N3)C (= O)NC4CCC5 = C4C = CC (= N5)N	-100.32	-9.96
***C11***	C_15_H_26_N_6_O	CN1CCCC1CCN2CCCC(C2)N3C = NC (= N3)C (= O)N	-70.92	-9.96
C12	C_19_H_27_N_7_O	C1CC1CN2CCN(CC2)C3 = CC = CC (= C3)NC (= O)C4 = CN(N = N4)CCN	-97.08	-9.92
C13	C_15_H_13_N_7_	CC1 = NC (= CN1C2N = CN = N2)C3 = CC = CC (= C3)C4 = CN = CN4	-72.17	-9.88
C14	C_16_H_29_N_7_O	CC1C(C(NN1)C)CNC (= O)C2 = CN(N = N2)CCC3CCCCN3	-66.18	-9.84
C15	C_19_H_30_N_6_O	CN1C (= NC (= N1)N)NCCCOC2 = CC = CC (= C2)CNC3CCCCC3	-83.69	-9.83
***C16***	C_19_H_25_N_5_O_2_	C1CC1NC (= O)C2 = CN(N = N2)CC3(CCN(CC3)CC4 = CC = CC = C4)O	-96.77	-9.78
***C17***	C_15_H_26_N_6_O	CN1CCCC1CCN2CCCC(C2)N3C = NC (= N3)C (= O)N	-68.92	-9.73
C18	C_23_H_32_N_6_O_2_	CN1CCC(CC1)CC (= O)N2CCCC2CN3C = C(N = N3)C (= O)NCC4 = CC = CC = C4	-105.47	-9.58
C19	C_15_H_25_N_5_O	CC1C(NNN1)C (= O)NCC(C2 = CC = C(C = C2)C)N(C)C	-70.90	-9.51
***C20***	C_15_H_26_N_6_O	CN1CCCC1CCN2CCCC(C2)N3C = NC (= N3)C (= O)N	-69.06	-9.51
C21	C_21_H_27_N_7_O_2_	C1CN(CCC1(CN2C = C(N = N2)C (= O)NCCN3C = CN = C3)O)CC4 = CC = CC = C4	-100.14	-9.49
C22	C_20_H_30_N_6_O_2_	C1CCN(C1)CCCC (= O)NC2CC(C = C2)CNC (= O)C3 = NNN = C3C4CC4	-90.86	-9.47
C23	C_23_H_34_N_6_O	CC(C)N1CCC(CC1)N2CCCC2CN3C = C(N = N3)C (= O)NCC4 = CC = CC = C4	-108.49	-9.45
C24	C_26_H_31_FN_6_O_3_	CC(C)C1 = C(C = C(C (= C1)C2 = NN = C(N2C3 = CC (= C(C = C3)N4CCN(CC4)C)F)C (= O)NC5CC5)O)O	-52.92	-9.45
C25	C_20_H_21_ClFN_5_O_2_	C1 = CC (= CC = C1CC(CC(CO)N)NC (= O)C2 = NNN = C2)C3 = C(C = CC (= C3)Cl)F	-92.16	-9.44
C26	C_18_H_27_N_7_OS	C1CCNC(C1)CCN2C = C(N = N2)C (= O)NCC3CNNC3C4 = CC = CS4	-85.28	-9.42
C27	C_23_H_32_FN_7_O	CCCCC(CC1 = CNC2 = CC = CC = C21)NC (= O)C3 = NC (= NN3)N4CCN(CC4)CCF	-91.04	-9.39
***C28***	C_15_H_26_N_6_O	CN1CCCC1CCN2CCCC(C2)N3C = NC (= N3)C (= O)N	-73.67	-9.39
C29	C_16_H_22_N_4_O_3_	C1COC2 = C1C = C(C = C2)C(C3CC(C3)O)NC (= O)C4CNNN4	-79.45	-9.38
C30	C_26_H_29_N_9_	C1CC2 = C(C (= CC (= N2)N3C (= NC (= N3)NC4 = CC5 = C(CCNC5)N = C4)N)C67CCC (= CC6)C = C7)NC1	-79.70	-9.37
C31	C_14_H_24_N_6_	C1CC2 = NC = NN2C1CN3CCC(C3)N4CCNCC4	-49.34	-9.37
C32	C_21_H_25_N_7_O_2_	CC1 = C(C = C(C = C1)C2 = NNC (= O)C = C2)NC (= O)C3 = CN(N = N3)CCC4CCCCN4	-97.57	-9.34
C33	C_8_H_16_N_4_S	CN1C(NN = C1C2CCCC2N)S	-55.17	-9.33
C34	C_20_H_23_ClN_6_O	CC1 = CC (= CC (= C1C2 = CC = C(C = C2)OCC3CCCN3)Cl)NC4 = NNC (= N4)N	-72.80	-9.31
C35	C_17_H_17_FN_6_O_2_	C1C(CN1)CN2C = C(N = N2)C (= O)NCC3 = CC4 = C(C = C(C = C4)F)NC3 = O	-84.83	-9.29
C36	C_18_H_22_N_6_O	CN1CCC(C1)N2C = C(N = N2)C (= O)N(C)CC3 = CC4 = C(C = C3)C = CN4	-77.46	-9.29
C37	C_21_H_26_N_6_O_3_	CC1 = C(C (= NO1)C)C2 = C(C = CC (= C2)NC (= O)C3 = NC = NN3C)OCC4CCCCN4	-75.26	-9.28
C38	C_22_H_31_FN_6_O_2_	C1CN(CCC1CNC (= O)C2 = CN(N = N2)CC3(CCNCC3)O)CC4 = CC = C(C = C4)F	-97.35	-9.22
***C39***	C_15_H_23_N_7_O	CC1 = NC = CN1CCCN2CCCC(C2)N3C = NC (= N3)C (= O)N	-70.34	-9.21
C40	C_21_H_25_N_5_O_3_	C1CN(CCN1CC2 = CC3 = C(C = C2)OCO3)C (= O)C4C(NNN4)C5 = CC = CC = C5	-84.38	-9.21
C41	C_21_H_22_ClN_7_O	CC1 = CC (= NC (= C1CN2C(C = NN2CC3 = CC4 = CC (= CN = C4C = C3)Cl)C (= O)N)C)N	-79.81	-9.21
C42	C_25_H_35_N_7_O	CC(C)CCC(CC1 = CNC2 = CC = CC = C21)NC (= O)C3 = NC (= NN3)N4CC5CCC(C4)N5C	-87.46	-9.20
C43	C_20_H_24_F_3_N_7_O	C1CCN(C1)CCOC2 = CC = C(C = C2)C3(NN(C (= N3)N)C4 = C(C = CC = N4)C(F)(F)F)N	-65.51	-9.20
C44	C_18_H_21_N_7_O_2_	C1CC(CNC1)CN2C = C(N = N2)C (= O)NCC3 = NC4 = CC = CC = C4C (= O)N3	-87.09	-9.19
C45	C_18_H_21_N_7_O_2_	C1CC(CNC1)CN2C = C(N = N2)C (= O)NCC3 = NC4 = CC = CC = C4C (= O)N3	-86.87	-9.19
C46	C_21_H_25_FN_6_OS	CN1CCN(CC1)CCCNC (= O)C2 = NN(C (= N2)C3 = CC = CS3)C4 = CC = C(C = C4)F	-76.37	-9.18
C47	C_17_H_28_N_6_O	C1CC(C2CCCN2C1)NC (= O)C3 = CN(N = N3)C4CCC(CC4)N	-80.11	-9.18
C48	C_17_H_24_N_6_O_2_	CCOC (= O)C1 = CN(N = N1)C2 = CC (= C(C = C2)N3CCCN(CC3)C)N	-61.09	-9.17
***C49***	C_14_H_23_N_5_O_2_	C1CCC(C1)N2CCC(CC2)CNC (= O)C3 = NNC (= O)N3	-71.44	-9.17
C50	C_15_H_19_FN_6_O_2_	CN1CCN(CC1)C2 = CC (= C(C = C2N)N3C = C(N = N3)C (= O)OC)F	-59.25	-9.14
C51	C_8_H_15_N_5_S	CCCN1CC(C1)N2C (= NNC2 = S)N	-41.51	-9.13
C52	C_22_H_32_N_6_O_2_	C1CCN(C1)CCNC (= O)C2 = CN(N = N2)CC3(CCN(CC3)CC4 = CC = CC = C4)O	-97.48	-9.13
C53	C_18_H_13_FN_4_O_3_	C1 = CC (= CC = C1C#CC2 = CC (= CC (= C2)F)OC3C(NNN3)C (= O)O)C#N	-60.06	-9.07
C54	C_20_H_25_N_7_O	CC1 = NC = CN1CC2 = CC = CC = C2NC (= O)C3 = CN(N = N3)CC4CCCNC4	-87.80	-9.06
C55	C_18_H_21_N_7_O_2_	C1CC(CNC1)CN2C = C(N = N2)C (= O)NCC3 = NC4 = CC = CC = C4C (= O)N3	-87.15	-9.05
C56	C_3_H_6_N_4_^+2^	C = [N+]1C = C[N+] (= N)N1	-38.32	-9.01
C57	C_17_H_22_N_6_O_2_	C1CC(NC1)CN2C = C(N = N2)C (= O)NC3 = CC = C(C = C3)CCC (= O)N	-86.14	-9.00
C58	C_20_H_25_N_7_O	CC1 = NC = CN1CC2 = CC = CC = C2NC (= O)C3 = CN(N = N3)C4CCC(CC4)N	-82.71	-8.99
C59	C_19_H_29_N_7_O	CN1CCN(CC1)C2 = CC = CC = C2CNC (= O)C3 = CN(N = N3)CCCCN	-84.01	-8.99
C60	C_21_H_29_N_5_O	CN1CCC2(CCC(CC2)NC (= O)C3 = CN = NN3CC4 = CC = CC = C4)CC1	-78.17	-8.97
C61	C_22_H_27_ClN_8_O	CN1C2 = CC = CC = C2N = C1N3C (= NC(N3)(C4 = CC (= C(C = C4)OCCN5CCCC5)Cl)N)N	-79.22	-8.94
***C62***	C_15_H_23_N_7_O	CC1 = NC = CN1CCCN2CCCC(C2)N3C = NC (= N3)C (= O)N	-72.35	-8.94
C63	C_18_H_24_N_6_O	C1CC(NC1)CN2C = C(N = N2)C (= O)NC3CNCC3C4 = CC = CC = C4	-82.92	-8.94
C64	C_19_H_30_N_8_O	CC1 = C(N = NN1C2 = NN(C = C2)C)C (= O)NC3CCN(CC3)C4CCN(CC4)C	-82.68	-8.93
C65	C_18_H_17_N_5_O	C1C(C(C2 = CC = CC = C21)N)NC (= O)C3 = CN(N = N3)C4 = CC = CC = C4	-76.70	-8.91
C66	C_16_H_19_N_3_O_3_	COC1 = CC (= CC (= C1)C2 = CC (= CC = C2)OC3CNNN3)OC	-63.37	-8.89
***C67***	C_16_H_18_N_6_O_2_	C1 = CC = C(C = C1)CCCN2C = CN = C2CNC (= O)C3 = NNC (= O)N3	-82.24	-8.86
C68	C_18_H_29_N_5_O	CC1C(NNN1)C (= O)NCC2CCCN(C2)CCC3 = CC = CC = C3	-77.89	-8.86
C69	C_17_H_31_N_7_O	CC1C(C(NN1)C)CCCNC (= O)C2 = CN(N = N2)C3CCC(CC3)N	-82.99	-8.85

This section may be divided by subheadings. It should provide a concise and precise description of the experimental results, their interpretation, as well as the experimental conclusions that can be drawn.

The selection of the best compounds was also based on the favorable values of ADMET properties obtained from the QikProp software. This software provides predictions for various parameters such as the octanol/water partition coefficient, skin permeability, human oral absorption percentage, and the number of likely metabolic reactions.

The predicted values for these properties were assessed and compared for the 81,197 triazole derivatives. The compounds that exhibited good values for these ADMET properties were shortlisted for further analysis and presented in **Table 2**. All 69 compounds showed good to moderate values of the analyzed parameters.

**Table 2:** Predicted values of ADMET properties using QikProp software, with the permissible values.

N	Volume	CIQPlogS	QPlogBB	metab	QPPMDCK	QPPCaco	QPlogPo/w	RuleOfFive	QPlogKp	% Human Oral Absorption
C1	1390.24	-2.39	-0.99	5	7.15	16.47	1.88	0	-7.60	59.71
C2	1292.92	-4.34	-1.27	2	36.38	47.02	2.60	0	-5.63	72.08
C3	1261.21	-3.41	-0.64	2	108.61	129.72	2.58	0	-5.23	79.84
C4	1212.87	-3.45	-0.83	6	50.64	3.54	1.88	0	-5.72	47.74
C5	1109.16	-5.08	-1.32	3	157.21	127.63	2.04	0	-4.35	76.56
C6	1059.70	-0.66	-0.20	1	12.30	27.20	0.49	0	-8.06	55.49
C7	1055.55	-2.26	-1.66	4	10.59	26.00	0.90	0	-5.96	57.55
C8	410.38	-0.05	-0.98	0	88.70	203.91	-1.45	0	-4.20	59.82
C9	1246.20	-3.36	-1.79	3	7.29	18.41	1.17	0	-6.89	56.45
C10	1432.10	-4.51	-1.31	5	28.96	65.93	3.01	0	-5.17	77.13
C11	1080.03	-0.12	-0.43	3	8.93	20.21	-0.01	0	-7.99	50.26
C12	1258.81	-1.63	-0.88	4	6.51	15.10	1.15	0	-7.55	54.76
C13	946.16	-3.78	-0.81	1	198.12	428.86	1.90	0	-3.05	85.17
C14	1163.56	0.03	-0.78	0	1.55	3.65	-0.05	0	-8.11	36.72
C15	1279.45	-3.56	-1.06	3	75.60	160.18	3.13	0	-4.36	84.70
C16	1215.14	-2.82	-0.86	3	66.01	141.28	2.37	0	-4.56	79.29
C17	1079.85	-0.12	-0.44	3	8.98	20.32	-0.01	0	-7.98	50.29
C18	1445.48	-2.87	-0.83	3	82.69	123.26	2.37	0	-4.37	78.25
C19	989.06	0.65	-0.08	4	3.77	5.63	-0.29	0	-7.31	38.67
C20	1079.27	-0.12	-0.44	3	8.91	20.17	-0.02	0	-7.99	50.20
C21	1364.81	-3.61	-1.44	3	30.92	70.04	2.39	0	-4.49	73.98
C22	1343.23	-2.04	-1.28	4	35.16	51.97	1.50	0	-5.53	66.45
C23	1416.91	-2.59	-0.03	2	39.42	79.87	2.92	0	-6.02	78.10
C24	1534.77	-5.43	-1.26	4	27.80	46.62	3.31	0	-6.08	76.16
C25	1244.79	-4.49	-1.48	4	34.75	25.28	2.16	0	-5.55	64.69
C26	1215.32	-1.25	-0.60	2	1.98	3.20	0.46	0	-7.98	38.69
C27	1373.32	-5.43	-0.48	2	174.37	200.17	4.09	0	-4.44	92.06
C28	1080.17	-0.12	-0.43	3	8.93	20.21	-0.01	0	-7.99	50.26
C29	1013.27	-0.50	-0.61	3	4.83	7.55	-0.29	0	-8.24	40.95
C30	1448.25	-5.48	-1.07	9	28.66	65.30	2.93	0	-5.56	76.57
C31	969.45	0.98	0.40	2	10.58	21.55	-0.08	0	-6.90	50.36
C32	1340.48	-4.06	-1.94	1	7.88	19.77	2.05	0	-6.31	62.13
C33	679.94	-0.92	0.30	3	424.03	348.64	0.75	0	-5.04	76.82
C34	1247.65	-4.65	-1.37	2	36.55	42.47	2.56	0	-5.62	71.10
C35	1106.91	-4.55	-1.57	1	121.26	157.38	2.49	0	-3.83	80.82
C36	1042.42	-2.95	-0.12	2	107.96	222.74	1.65	0	-4.76	78.60
C37	1342.16	-4.01	-1.11	3	31.02	70.26	2.44	0	-5.59	74.27
C38	1394.43	-2.86	-0.62	3	21.44	26.27	2.24	0	-6.90	65.44
C39	1080.35	-1.80	-1.12	3	21.61	50.28	0.67	0	-5.91	61.30
C40	1198.68	-1.17	0.21	5	8.01	12.96	0.70	0	-6.16	50.95
C41	1251.81	-3.45	-1.11	7	37.21	18.73	0.97	0	-5.65	55.39
C42	1494.10	-5.58	-0.61	2	126.99	258.83	4.77	0	-4.11	100.00
C43	1283.49	-2.02	-0.34	4	7.92	6.87	0.94	0	-6.82	47.44
C44	1185.51	-2.66	-1.60	1	10.28	25.29	0.64	0	-6.24	55.79
C45	1185.51	-2.66	-1.60	1	10.28	25.29	0.64	0	-6.24	55.79
C46	1368.05	-3.27	0.11	3	102.08	67.06	2.95	0	-6.09	76.91
C47	1123.77	-1.02	-0.37	1	8.59	19.51	0.67	0	-8.21	53.98
C48	1165.39	-2.35	-0.85	3	37.64	84.02	1.74	0	-5.72	71.59
C49	1000.04	-2.18	-1.31	0	9.64	23.82	1.14	0	-7.19	58.26
C50	1059.69	-2.59	-0.74	2	45.52	66.43	1.14	0	-6.12	66.21
C51	730.22	-2.03	-0.55	0	664.50	613.26	1.28	0	-3.55	84.33
C52	1404.45	-2.36	-0.59	4	19.10	40.86	2.23	0	-6.29	68.83
C53	1093.86	-3.65	-1.74	1	0.37	0.49	-0.30	0	-8.82	19.66
C54	1261.40	-3.69	-0.99	3	42.33	93.67	2.46	0	-4.89	76.66
C55	1181.00	-2.66	-1.53	1	10.91	26.72	0.64	0	-6.20	56.22
C56	395.45	-0.71	-0.30	1	577.24	1153.44	-0.04	0	-2.69	81.54
C57	1150.06	-1.65	-2.01	3	5.05	6.40	-0.22	0	-6.76	40.11
C58	1206.36	-3.76	-0.78	4	42.94	94.92	2.22	0	-4.98	75.32
C59	1276.19	-1.86	-0.99	4	5.94	13.86	1.22	0	-7.53	54.54
C60	1238.19	-3.71	-0.09	2	222.42	434.72	3.51	0	-3.87	94.73
C61	1403.52	-2.39	-0.32	2	9.26	9.04	1.51	0	-6.48	52.93
C62	1077.66	-1.80	-1.20	3	17.63	41.65	0.58	0	-6.10	59.33
C63	1157.13	-1.62	-0.34	2	12.80	28.21	1.19	0	-7.11	59.89
C64	1301.13	-1.65	0.12	2	31.46	64.83	1.57	0	-6.99	68.56
C65	1050.89	-3.37	-0.70	3	46.41	101.99	2.19	0	-4.58	75.71
C66	973.51	-1.63	0.16	2	37.09	75.49	1.09	0	-6.17	66.93
C67	1067.97	-4.71	-2.11	2	34.49	85.10	2.58	0	-3.83	76.60
C68	1164.89	-0.15	-0.41	3	3.36	4.89	0.81	0	-6.93	44.03
C69	1218.85	-0.24	-1.23	1	0.64	1.60	0.00	0	-8.79	30.62

• Volume: Total solvent-accessible volume in cubic Ångströms using a probe with a 1.4Å radius (500.0–2000.0)

• QPlogPo/w: Predicted octanol/water partition coefficient (–2.0–6.5)

• QplogS: (−6.5 to 0.5)

• QPlogBB: Predicted brain/blood partition coefficient (–3.0–1.2)

• QPloghERG: (<−5)

• QPlogKp: Predicted skin permeability (–8.0–1.0)

• QPPCaco ‐ Predicted Caco-2 cell permeability in nm/sec, (<25 poor, >500 great)

• QPPMDCK ‐ Predicted MDCK cell permeability, (<25 poor, >500 great)

• #metabol: The number of likely metabolic reactions (1–8)

• HOA (%) ‐ Predicted human oral absorption in percentage (0–100 scale), > 80% high, < 25% low

• Rule of five ‐ Number of violations from Lipinski’s rule of five.

### ADMET analysis

The ADMET properties of the top 69 compounds were analyzed using SwissADME-Tox, and PEO software. From this analysis, nine compounds (C11, C16, C17, C20, C28, C39, C49, C62, and C67) were identified as having favorable parameters for potential oral medication (**Table 3)**.

**Table 3:** ADMET parameters prediction of the best selected compounds using SwissAdme-Tox, Osiris Property Explorer, and docking score.

Molecule	C11	C16	C17	C20	C28	C39	C49	C62	C67
**MW**	306.41	355.43	306.41	306.41	306.41	317.39	293.36	317.39	326.35
**Heavy atoms**	22	26	22	22	22	23	21	23	24
**Aromatic heavy atoms**	5	11	5	5	5	10	5	10	16
**Fraction Csp3**	0.8	0.53	0.8	0.8	0.8	0.6	0.79	0.6	0.25
**Rotatable bonds**	5	7	5	5	5	6	5	6	8
**H-bond acceptors**	5	5	5	5	5	5	4	5	4
**H-bond donors**	1	2	1	1	1	1	3	1	3
**MR**	91.65	101.01	91.65	91.65	91.65	89.26	82.86	89.26	87.84
**TPSA**	80.28	83.28	80.28	80.28	80.28	94.86	93.88	94.86	108.46
**iLOGP**	2.62	2.85	2.72	2.7	2.78	2.05	2	2.03	1.66
**XLOGP3**	0.66	1.01	0.66	0.66	0.66	0.19	0.96	0.19	0.77
**WLOGP**	-0.26	0.6	-0.26	-0.26	-0.26	0.23	0.1	0.23	0.71
**MLOGP**	0.62	1.32	0.62	0.62	0.62	0.02	1.08	0.02	0.77
**Silicos-IT Log P**	-0.21	1.33	-0.21	-0.21	-0.21	-0.25	1.57	-0.25	2.17
**Consensus Log P**	0.69	1.42	0.71	0.7	0.72	0.45	1.14	0.44	1.21
**ESOL Log S**	-1.99	-2.53	-1.99	-1.99	-1.99	-1.85	-2.11	-1.85	-2.31
**Solubility**	Very	Soluble	Very	Very	Very	Very	Soluble	Very	Soluble
**GI absorption**	High	High	High	High	High	High	High	High	High
**BBB permeant**	No	No	No	No	No	No	No	No	No
**P-gp substrate**	Yes	Yes	Yes	Yes	Yes	Yes	Yes	Yes	Yes
**CYP1A2 inhibitor**	No	No	No	No	No	No	No	No	No
**CYP2C19 inhibitor**	No	No	No	No	No	No	No	No	No
**CYP2C9 inhibitor**	No	No	No	No	No	No	No	No	No
**CYP2D6 inhibitor**	No	No	No	No	No	No	No	No	No
**CYP3A4 inhibitor**	No	No	No	No	No	No	No	No	No
**log Kp (cm/s)**	-7.7	-7.75	-7.7	-7.7	-7.7	-8.1	-7.41	-8.1	-7.74
**Lipinski violations**	0	0	0	0	0	0	0	0	0
**Ghose violations**	0	0	0	0	0	0	0	0	0
**Veber violations**	0	0	0	0	0	0	0	0	0
**Egan violations**	0	0	0	0	0	0	0	0	0
**Muegge violations**	0	0	0	0	0	0	0	0	0
**Bioavailability Score**	0.55	0.55	0.55	0.55	0.55	0.55	0.55	0.55	0.55
**PAINS alerts**	0	0	0	0	0	0	0	0	0
**Brenk alerts**	0	0	0	0	0	0	0	0	0
**Leadlikeness violations**	0	1	0	0	0	0	0	0	1
**Synthetic Accessibility**	3.79	3.11	3.79	3.79	3.79	3.48	3.33	3.48	2.56
**docking score (Kcal/Mol)**	-9.96	-9.78	-9.73	-9.51	-9.39	-9.21	-9.16	-8.94	-8.86
**Toxicity**	No indication for mutagenicity, tumorigenicity, irritating or reproductive effect								
**drug score**	0.95	0.9	0.95	0.95	0.95	0.95	0.92	0.95	0.93

These compounds demonstrated high to moderate solubility, indicating their ability to dissolve well in biological fluids. This is important for effective administration. They also showed high gastrointestinal (GI) absorption, suggesting easy absorption into the bloodstream through the gastrointestinal tract, thus increasing their potential bioavailability. Additionally, these compounds have the potential to be transported by P-glycoprotein (P-gp), which can affect their distribution and elimination.

Furthermore, these compounds were predicted not to inhibit specific CYP enzymes (CYP1A2, CYP2C19, CYP2C9, CYP2D6, CYP3A4). This is favorable because it means they are less likely to interfere with the metabolism of other drugs. They were also found to have limited blood-brain barrier (BBB) permeability, indicating a reduced ability to cross the BBB. This can be advantageous depending on their intended targets.

Moreover, these compounds exhibited high drug scores, indicating desirable properties for potential therapeutic use, as well as good synthetic accessibility, which means they can be feasibly synthesized in a laboratory setting. They also did not show any presence of pains or Brenk alerts, suggesting the absence of substructures associated with potential toxicity or undesirable properties. Further, all selected compounds adhered to the rules of lead-likeness, indicating characteristics commonly associated with drug-like molecules. Finally, the selected compounds are predicted to not have any indication for mutagenicity, tumorigenicity, irritating or reproductive effect.

In summary, these findings suggest that the nine selected compounds (C11, C16, C17, C20, C28, C39, C49, C62, and C67) have favorable ADMET properties and may be promising candidates for further development as potential oral medications.

### The molecular interactions of the best selected compounds complexed with target

**Fig 3:**
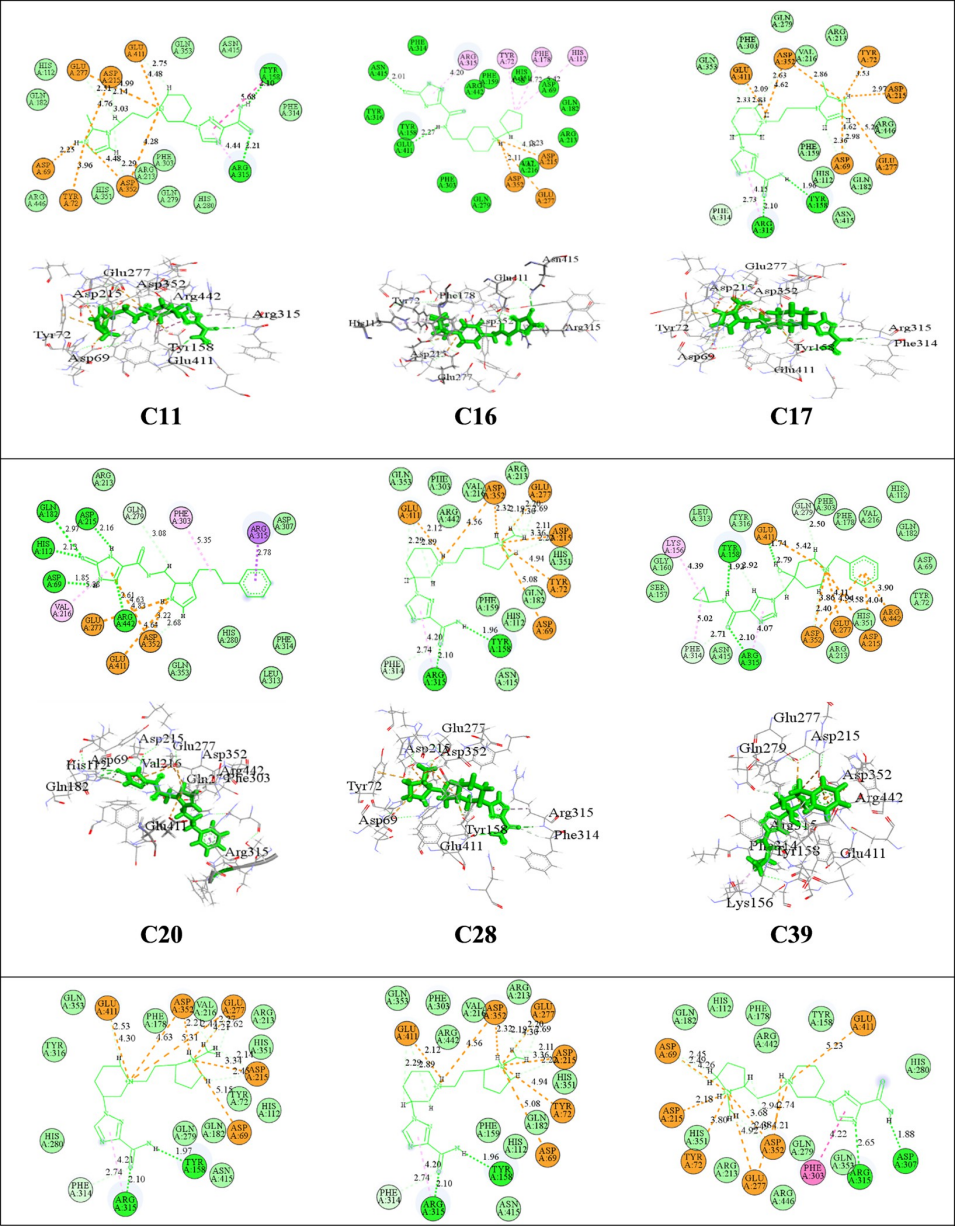
The 2D and 3D visualization of created complexes with the main residues, and interaction type.

In the complexes formed between the ligands (compounds C11, C16, C17, C20, C28, C39, C49, C62, and C67) and the target, various molecular interactions play a crucial role in their stability and binding affinity (**Fig 3** and **Table 4**).

**Table 4:** The molecular interaction type between each docked ligand and key residues of target, with their distances expressed in Ångströms (Å).

N	Residue	Distance (Å)	Interaction type	N	Residues	Distance (Å)	Interaction type
**C67**	Arg315	2.78	Pi-Sigma	**C20**	Arg315	2.87	Conventional Hydrogen
	Arg442	2.61	Conventional Hydrogen		Asp215	2.11	Salt Bridge; Attractive Charge
	Asp215	2.16	Conventional Hydrogen		Asp215	3.06	Carbon Hydrogen Bond
	Asp352	3.22	Salt Bridge; Attractive Charge		Asp352	3.86	Attractive Charge
	Asp352	2.68	Carbon Hydrogen Bond		Asp352	3.68	Attractive Charge
	Asp352	4.63	Pi-Anion		Asp352	2.42	Carbon Hydrogen Bond
	Asp69	1.85	Conventional Hydrogen		Asp352	2.81	Carbon Hydrogen Bond
	Gln182	2.97	Conventional Hydrogen		Asp352	2.43	Carbon Hydrogen Bond
	Gln279	3.08	Carbon Hydrogen Bond		Asp352	2.43	Carbon Hydrogen Bond
	Glu277	4.83	Attractive Charge		Asp69	4.32	Attractive Charge
	Glu411	4.64	Attractive Charge		Asp69	2.35	Carbon Hydrogen Bond
	His112	2.13	Conventional Hydrogen		Glu277	4.2	Attractive Charge
	His112	3	Carbon Hydrogen Bond		Glu277	4.83	Attractive Charge
	Phe303	5.35	Pi-Alkyl		Glu411	5.25	Attractive Charge
	Val216	5.28	Pi-Alkyl		Glu411	2.78	Carbon Hydrogen Bond
**C39**	Arg315	2.21	Conventional Hydrogen		Tyr158	1.91	Conventional Hydrogen
	Arg315	4.44	Pi-Alkyl		Tyr158	4.87	Pi-Pi T-Shaped
	Asp215	4.76	Attractive Charge		Tyr72	3.88	Pi-Cation
	Asp215	2.14	Carbon Hydrogen Bond		Tyr72	2.16	Pi-Sigma
	Asp215	3.03	Carbon Hydrogen Bond	**C17**	Arg315	2.1	Conventional Hydrogen
	Asp352	4.28	Attractive Charge		Arg315	4.21	Pi-Alkyl
	Asp352	4.48	Attractive Charge		Asp215	3.34	Attractive Charge
	Asp352	2.29	Carbon Hydrogen Bond		Asp215	2.45	Carbon Hydrogen Bond
	Asp69	2.25	Salt Bridge; Attractive Charge		Asp215	2.14	Carbon Hydrogen Bond
	Glu277	4.99	Attractive Charge		Asp352	2.21	Salt Bridge; Attractive Charge
	Glu277	2.51	Carbon Hydrogen Bond		Asp352	4.63	Attractive Charge
	Glu411	4.48	Attractive Charge		Asp352	2.44	Carbon Hydrogen Bond
	Glu411	2.75	Carbon Hydrogen Bond		Asp69	5.15	Attractive Charge
	Tyr158	2.1	Conventional Hydrogen		Glu277	5.31	Attractive Charge
	Tyr158	5.68	Pi-Pi T-Shaped		Glu277	4.21	Attractive Charge
	Tyr72	3.96	Pi-Cation		Glu277	2.27	Carbon Hydrogen Bond
**C28**	Arg315	2.65	Conventional Hydrogen		Glu277	2.62	Carbon Hydrogen Bond
	Asp215	2.18	Salt Bridge; Attractive Charge		Glu411	4.3	Attractive Charge
	Asp307	1.88	Conventional Hydrogen		Glu411	2.53	Carbon Hydrogen Bond
	Asp352	2.74	Salt Bridge; Attractive Charge		Phe314	2.74	Carbon Hydrogen Bond
	Asp352	3.68	Attractive Charge		Tyr158	1.97	Conventional Hydrogen
	Asp352	2.94	Carbon Hydrogen Bond	**C11**	Arg315	2.1	Conventional Hydrogen
	Asp352	2.38	Carbon Hydrogen Bond		Arg315	4.2	Pi-Alkyl
	Asp352	2.49	Carbon Hydrogen Bond		Asp215	3.36	Attractive Charge
	Asp69	4.26	Attractive Charge		Asp215	2.22	Carbon Hydrogen Bond
	Asp69	2.49	Carbon Hydrogen Bond		Asp215	2.11	Carbon Hydrogen Bond
	Asp69	2.45	Carbon Hydrogen Bond		Asp352	2.32	Salt Bridge; Attractive Charge
	Glu277	4.21	Attractive Charge		Asp352	4.56	Attractive Charge
	Glu277	4.92	Attractive Charge		Asp352	2.19	Carbon Hydrogen Bond
	Glu411	5.23	Attractive Charge		Asp69	5.08	Attractive Charge
	Phe303	4.22	Pi-Pi Stacked		Glu277	4.3	Attractive Charge
	Tyr72	3.8	Pi-Cation		Glu277	2.2	Carbon Hydrogen Bond
**C62**	Arg315	2.1	Conventional Hydrogen		Glu277	2.69	Carbon Hydrogen Bond
	Arg315	4.15	Pi-Alkyl		Glu411	2.12	Salt Bridge; Attractive Charge
	Asp215	2.97	Salt Bridge; Attractive Charge		Glu411	2.29	Carbon Hydrogen Bond
	Asp352	4.62	Attractive Charge		Glu411	2.89	Carbon Hydrogen Bond
	Asp352	2.86	Attractive Charge		Phe314	2.74	Carbon Hydrogen Bond
	Asp352	2.63	Carbon Hydrogen Bond		Tyr158	1.96	Conventional Hydrogen
	Asp69	4.62	Attractive Charge		Tyr72	4.94	Pi-Cation
	Asp69	2.36	Carbon Hydrogen Bond	**C16**	Arg315	2.1	Conventional Hydrogen
	Asp69	2.98	Carbon Hydrogen Bond		Arg315	4.07	Pi-Alkyl
	Glu277	5.26	Attractive Charge		Arg442	3.9	Pi-Cation
	Glu411	2.09	Salt Bridge; Attractive Charge		Asp215	5.58	Attractive Charge
	Glu411	2.33	Carbon Hydrogen Bond		Asp215	4.04	Pi-Anion
	Glu411	2.83	Carbon Hydrogen Bond		Asp352	3.86	Attractive Charge
	Phe314	2.73	Carbon Hydrogen Bond		Asp352	2.4	Carbon Hydrogen Bond
	Tyr158	1.96	Conventional Hydrogen		Asp352	4.94	Pi-Anion
	Tyr72	3.53	Pi-Cation		Gln279	2.5	Carbon Hydrogen Bond
**C49**	Arg315	4.2	Pi-Alkyl		Glu277	4.11	Attractive Charge
	Asn415	2.01	Conventional Hydrogen		Glu411	5.42	Attractive Charge
	Asp215	4.18	Attractive Charge		Glu411	1.74	Conventional Hydrogen
	Asp215	2.23	Carbon Hydrogen Bond		Glu411	2.79	Carbon Hydrogen Bond
	Asp352	2.11	Salt Bridge; Attractive Charge		Lys156	4.39	Alkyl
	Glu277	4.17	Attractive Charge		Phe314	2.71	Carbon Hydrogen Bond
	Glu411	2.27	Conventional Hydrogen		Phe314	5.02	Pi-Alkyl
	His112	5.42	Pi-Alkyl		Tyr158	1.92	Conventional Hydrogen
	Phe178	4.72	Pi-Alkyl		Tyr158	2.92	Carbon Hydrogen Bond
	Tyr72	4.58	Pi-Alkyl				

Conventional hydrogen bonds are observed in all complexes, involving residues such as Arg315, Asp215, Asp69, Gln182, Glu411, His112, and Tyr158 with a range of 1.74–2.97 Å. These hydrogen bonds contribute to the overall stability of the ligand-target complexes. Carbon hydrogen bonds also contribute to the binding in all complexes, involving residues Asp215, Asp352, Gln79, Glu277, Glu411, His112, and Phe314. These interactions further enhance the binding between the ligands and the target. Attractive charge interactions occur in all complexes, involving residues Asp215, Asp352, Asp69, Glu277, Glu411, and Tyr72 which are ranging between (2.09–5.58Å). These attractive charges contribute to the electrostatic interactions between the ligands and the target, further stabilizing the complexes.

Pi-alkyl interactions are observed in several complexes, including C11, C16, C17, C39, C49, C62, and C67, specifically involving residues Arg315, Phe303, and Val216. These interactions involve the stacking of aromatic rings and contribute to the overall binding affinity. Pi-cation interactions occur between the ligands and the residue Tyr72 in multiple complexes, such as C11, C16, C20, C28, C39, and C62. These interactions involve the interaction of a positively charged moiety with an aromatic ring, contributing to the stability of the ligand-target complexes.

Pi-Pi T-Shaped interactions are observed in complexes C16, C20, C28, and C39. These interactions involve the stacking of aromatic rings between the ligands and residues Phe303, Tyr158, and Tyr72. Pi-Pi T-Shaped interactions play a crucial role in the binding affinity and specificity of the ligands.

Finally, the Pi-Sigma interaction is exhibited with the Arg315 residue of the target in the C20 and C67 complexes, respectively. The Pi-Sigma interaction occurs when the pi system of the ligand interacts with the sigma bond of the target residue, forming a stabilizing interaction.

### Molecular dynamics simulation

**Fig 4:**
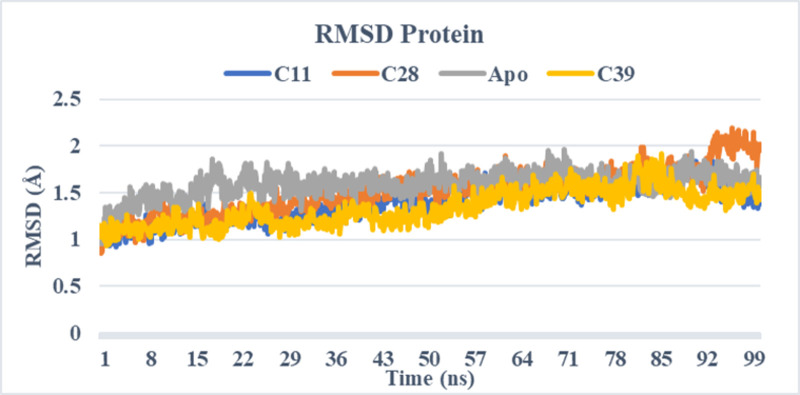
RMSD protein of simulated complexes (C11, C28, C39, and Apo-protein).

**Fig 5:**
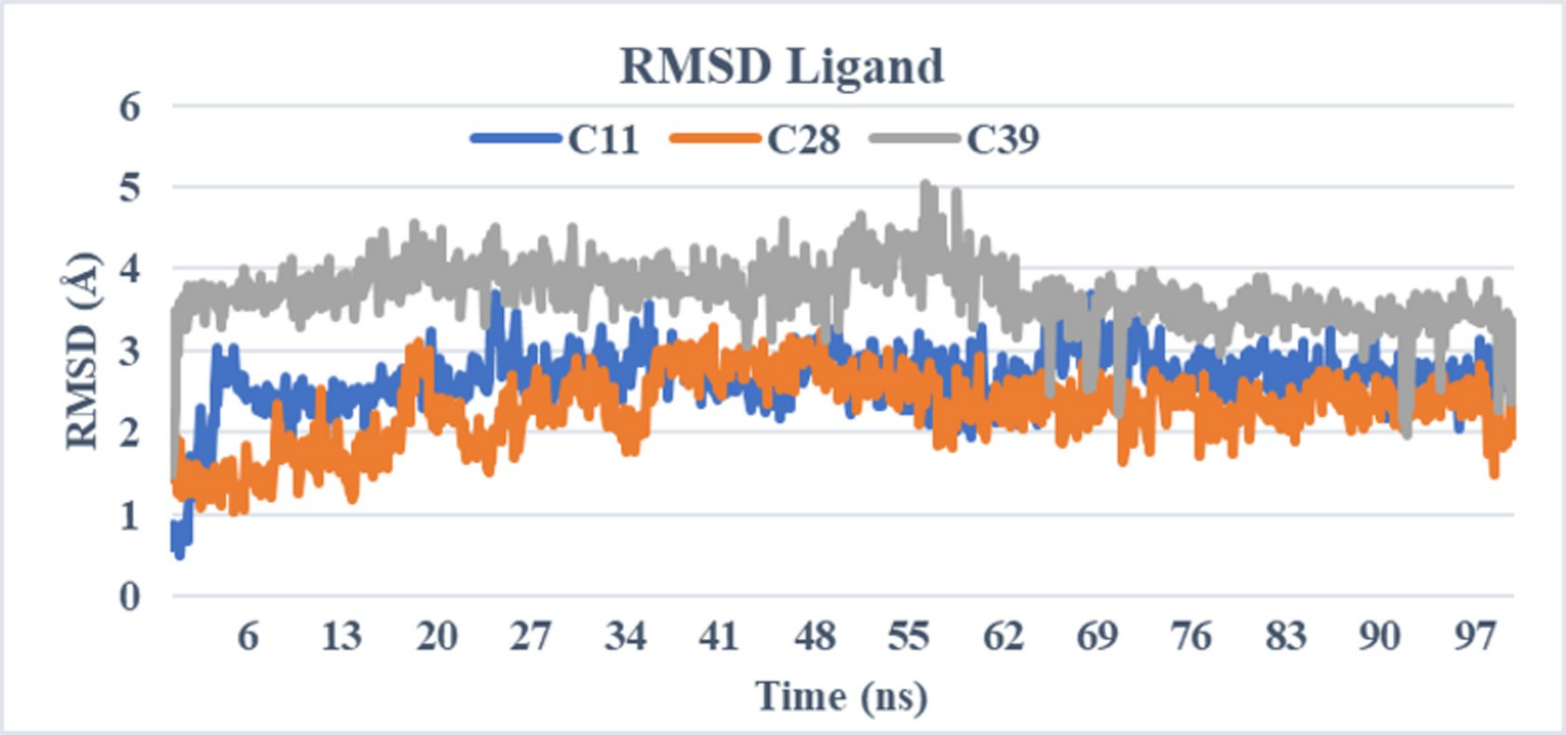
The RMSD ligand of simulated complexes (C11-3a4a, C28-3a4a, and C39-3a4a).

**Fig 6:**
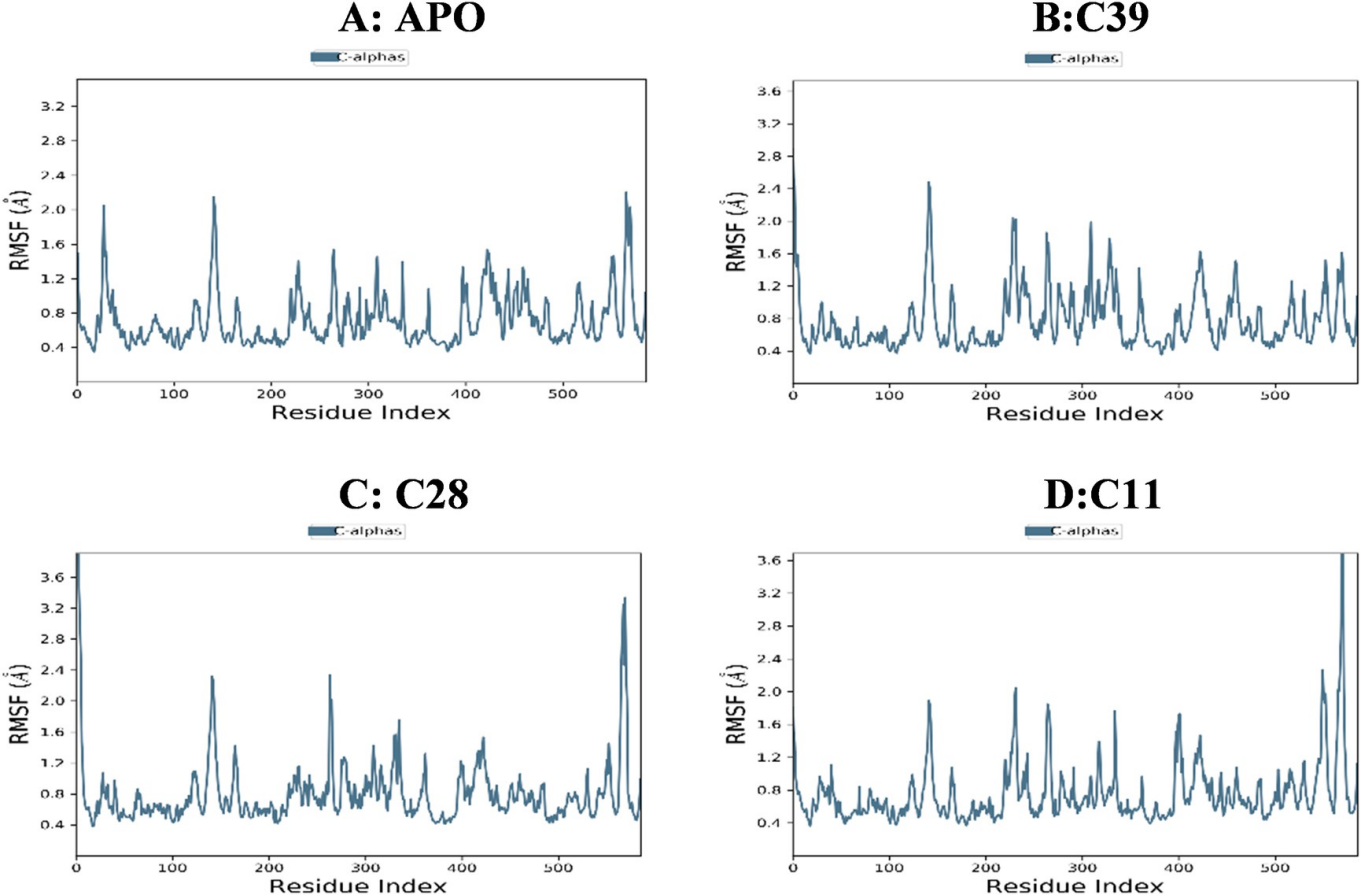
RMSF protein of all simulated complex (A: Apo-Protein, B:C39-3a4a, C:C28-3a4a, and D:C11-3a4a).

**Fig 7:**
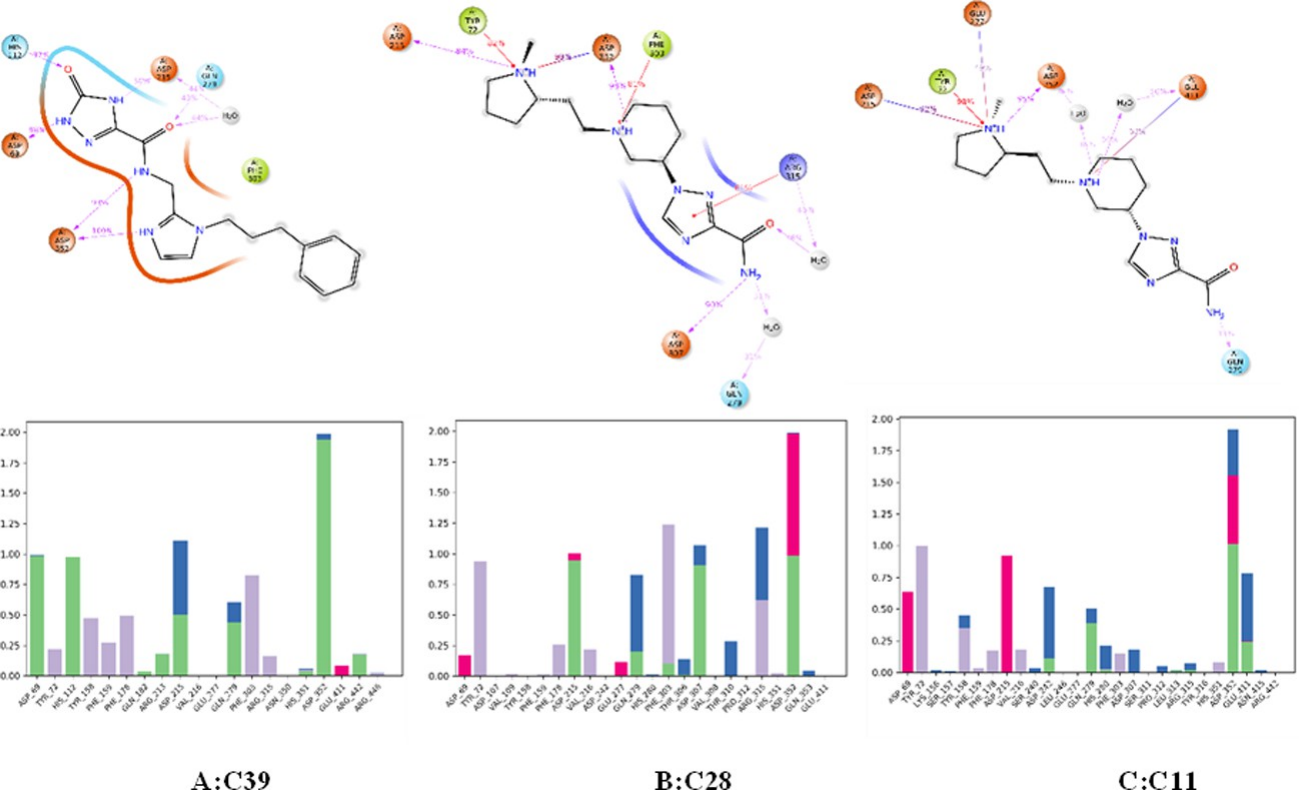
2D structure, and ligand-Protein contact of the created complexes (A: C39, B:C28, C:C11).

The unbound protein 3A4A and the top-selected compounds C11, C28, and C39 were analyzed using molecular dynamic simulations to assess the stability of the resulting complexes and detect any structural alterations in the protein or ligands. To gain a better understanding, a range of parameters such as RMSD, RMSF, ligand characteristics, and ligand-protein interactions were computed and visualized in **Figs 4–7**. These calculations and graphical representations offer valuable insights into the stability and behavior of the complexes.

#### Root mean square deviation

The stability of the protein-ligand complexes (C11, C28, and C39) and the un-complexed protein was assessed by calculating and comparing the root mean square deviation values. The RMSD values measure the deviation of the protein structure from its initial conformation during the molecular dynamic’s simulation. **Fig 4** displays the RMSD plots for each complexed compound and the apo protein.

Upon analysis, it was observed that all the protein-ligand complexes exhibited stability throughout the simulation, with minor fluctuations. The RMSD values for the complexes remained below 2.5 Å, indicating that there were no significant structural changes in the proteins despite their interaction with the ligands.

These findings suggest that the formed complexes maintained their overall structural integrity and stability throughout the simulation period. The minor fluctuations observed can be attributed to the inherent dynamics of the protein-ligand system.

The stability of the protein-ligand complexes was assessed through molecular dynamic simulations, and the results were analyzed using various parameters. The RMSD (root mean square deviation) values of the protein and ligands were calculated and plotted to evaluate any structural changes (**Fig 5**).

For the protein RMSD, all complexes (C11, C28, and C39) and the apo protein showed stability throughout the simulation, with minor fluctuations observed in the C28 complex towards the end of the simulation. The RMSD values for all complexes remained below 2.5 Å, indicating the absence of significant structural changes in the proteins despite their complexation with the ligands.

Regarding the ligand RMSD, the C11 and C28 complexes exhibited stability throughout the entire simulation, with no significant changes observed in their structures. However, the C39 ligand showed major fluctuations in its RMSD, exceeding 3 Å during the first 60 ns of the simulation. Afterward, the ligand achieved stability by the end of the simulation.

#### Root mean square fluctuation

The protein RMSF values were calculated for all simulated complexes, and the results were plotted in **Fig 6**. The RMSF values provide insights into the fluctuations in protein structure during the simulations. Minor fluctuations were observed in the protein structure for most of the complexes, indicating stability. However, the complex C11-3A4A exhibited a significant fluctuation that exceeded 3Å, suggesting a notable change in protein structure.

#### Ligand-protein contact

In the simulated complexes, multiple interactions were observed between the ligands and the target protein (**Fig 7**). In the complex C39-3a4a, the ligand C39 formed hydrogen bonds with Asp69, His112, and Asp352 residues, hydrophobic bonds with Tyr158, Phe178, and Phe303, water bridges with Asp215 and Gln279, and an ionic bond with Glu411. Similarly, in the complex formed by compound C28 and 3a4a, interactions included hydrogen bonds with Asp215, Asp307, and Asp352, hydrophobic bonds with Tyr72, Phe303, and Arg315, ionic bonds with Asp69, Glu277, and Asp352, and water bridges with Gln279, Thr310, and Arg315.

In the case of the complex C11-3a4a, interactions were observed, such as ionic bonds with Asp69, Asp215, and Asp352, hydrogen bonds with Gln279, Asp352, and Glu411, hydrophobic bonds with Tyr72, Tyr158, Phe178, and Val216, and water bridges with Asp242, Asp352, and Glu411. These interactions play a crucial role in stabilizing the protein-ligand complexes and influencing their binding affinity and specificity. The specific residues involved contribute to the overall stability and functionality of the complexes.

## Discussion

The present study employed a systematic approach combining computational and analytical methods to identify potential drug candidates targeting alpha-glucosidase, a key enzyme implicated in metabolic disorders such as type 2 diabetes mellitus. The research workflow involved multiple stages, beginning with the application of Lipinski’s Rule of Five to filter a vast library of compounds retrieved from the PubChem database. This initial step ensured that selected compounds exhibited favorable pharmacokinetic properties essential for oral drug delivery, thereby enhancing their potential efficacy and safety as therapeutic agents.

Following the Lipinski pre-filter, molecular docking simulations were conducted in three successive stages: High Throughput Virtual Screening (HTVS), Standard Precision (SP), and Extra Precision (XP) docking. These steps progressively refined the compound selection by evaluating binding affinities and interactions with alpha-glucosidase. The hierarchical docking strategy effectively narrowed down the initial compound pool to 69 promising candidates, based on robust scoring metrics and reliable binding poses.

Further validation through ADMET analysis using QikProp and SwissADME-Tox highlighted nine compounds (C11, C16, C17, C20, C28, C39, C49, C62, and C67) with optimal pharmacokinetics profiles suitable for oral administration. These compounds exhibited desirable properties such as high solubility, gastrointestinal absorption, minimal inhibition of key metabolic enzymes (CYP450), and limited blood-brain barrier permeability. Such characteristics are crucial for ensuring effective drug absorption, distribution, metabolism, excretion, and reduced toxicity risks, thereby reinforcing their candidacy for clinical development.

The molecular interactions between the selected compounds and alpha-glucosidase were analyzed through detailed docking studies and molecular dynamics (MD) simulations. These simulations revealed stable binding configurations and structural integrity of the complexes (C11, C28, and C39) over a 100 ns period, as evidenced by low RMSD values. Although minor fluctuations were observed in RMSF analysis, indicating localized protein flexibility, overall, the complexes maintained stability, which is indicative of strong ligand-protein interactions crucial for therapeutic efficacy.

The observed molecular interactions, including hydrogen bonds, pi-alkyl, pi-cation, and hydrophobic interactions, underscored the specificity and strength of ligand binding to the target enzyme. These interactions contribute significantly to the stability and binding affinity of the complexes, highlighting the potential of the identified compounds as effective alpha-glucosidase inhibitors.

## Conclusions

The analysis of 81,197 triazole derivatives revealed that these nine compounds demonstrate potent inhibitory activity against alpha-glucosidase, a critical target in diabetes treatment. They possess the necessary characteristics for further development and have shown promising results in both docking studies and molecular dynamics simulations. Initially, 69 compounds out of the total screened demonstrated significant binding affinity to alpha-glucosidase. Following ADMET analysis, these nine compounds emerged with desirable pharmacokinetic profiles, solidifying their candidacy as alpha-glucosidase inhibitors.

Detailed insights into the molecular interactions and dynamics of these triazole derivatives underscore their potential as pharmacological agents, particularly in inhibiting alpha-glucosidase. To validate their efficacy and safety as potential anti-diabetic drugs, rigorous in vitro and in vivo studies are recommended. These comprehensive assessments will deepen our understanding of their therapeutic potential and pave the way for their future development, advancing the field of rational drug design and precision medicine.
