# Supplementary material for: Exploration of alpha-glucosidase inhibitors: A comprehensive in silico approach targeting a large set of triazole derivatives
Source: PLoS One. 2024 Sep 6;19(9):e0308308. doi: 10.1371/journal.pone.0308308 (PMC11379377; doi:10.1371/journal.pone.0308308)
Supplement: Attachment — Submitted filename: responses to reviewers.docx [file pone.0308308.s001.docx]

*We would like to sincerely thank the reviewers for their valuable feedback and constructive comments, which have greatly contributed to improving the quality of this manuscript.*

Reviewer #1: Comments on PONE-D-24-10951

Overall, this regular manuscript entitled “Exploration of alpha-glucosidase inhibitors: A comprehensive in silico approach targeting a large set of triazole derivatives" for PLOS One by Abchir et al. is well written. However, the manuscript needs substantial revision in order to appreciate the quality before it can be considered for publication.

Comments:
1-Give a list of abbreviations.

The list of abbreviations has been added as recommended

2-The abstract should provide a concise summary of the key findings of the study.

The abstract has been rewritten to provide a concise summary of the key findings of the study, addressing the reviewer's suggestions.

3- Introduction section should be enriched by adding information on glucosidase inhibitors.

Additional information on alpha-glucosidase inhibitors has been incorporated into the Introduction section to enhance clarity and relevance.

4-Why did the author choose the targeted alpha-glucosidase inhibitors? Give your appropriate reasons separately.

In my thesis titled "Designing novel diabetes drugs using computational methods," we previously focused on alpha-amylase inhibitors as a treatment for diabetes mellitus. Now, we aim to explore alpha-glucosidase inhibitors.

- Alpha-glucosidase inhibitors are pivotal in managing type 2 diabetes by slowing carbohydrate breakdown in the small intestine, thereby regulating blood glucose levels.
- Clinical success, notably with acarbose, underscores their efficacy and supports further research and optimization of these inhibitors.
- The well-characterized structure of alpha-glucosidase makes it an ideal target for structure-based drug design, facilitating the development of potent inhibitors (resolution).
- There is a continuous need for new and more effective alpha-glucosidase inhibitors with fewer side effects. Investigating this target can lead to the discovery of new compounds with improved therapeutic profiles.

5- How do authors validate the protein? Should be added Ramachandran plot

The protein was validated using multiple criteria including R-free value, Clash-score, and Ramachandran plot analysis, all of which met established standards. The Ramachandran plot has been added and interpreted in the manuscript to provide clarity on the validation process

6. Figures 3 and 4 must be improved and modified for easily readable with resolution.

Figures 3 and 4 have been improved and modified to enhance readability and resolution, addressing the reviewer's feedback.

7- The discussion part is poor and must be refined and added with relevant work.

The Discussion section has been refined and expanded to include relevant literature and discuss the findings comprehensively, as suggested by the reviewer.

8- The conclusion part is not good to separate and indicates your goal of the study clearly.

The Conclusion section has been revised.

9- The following literature is recommended to be cited in the manuscript:

<https://doi.org/10.1371/journal.pone.0273256>
<https://doi.org/10.1016/j.molstruc.2024.137930>
<https://doi.org/10.1016/j.jsps.2023.101804>
<https://doi.org/10.1080/07391102.2023.2258404>
<https://doi.org/10.3390/molecules26227016>

The recommended literature has been cited in the manuscript to enrich the discussion and support the study's context and findings.

10- Many typo mistakes are there, check them thoroughly.

The manuscript has been thoroughly checked and typos have been corrected as per the reviewer's suggestion.

11-In the manuscript, there are also grammatical errors. Grammatical and punctuation errors must be corrected. Examine the manuscript thoroughly and improve the English language.

Grammatical and punctuation errors have been carefully reviewed and corrected to improve the overall clarity and readability of the manuscript.

My Opinion: Minor revision.

----------------------------------------------------------------------------------------------------------------

Reviewer #2: In this manuscript authors have performed virtual screening of 81,197 molecules to discover novel alpha-glucosidase inhibitors. Various insilico techniques have been used for virtual screening. The insilico studies without supporting invitro/invivo data have its limitations. In such cases previous supporting literature should be discussed precisely with proper citation. Following are my recommendations that needs to be included in revise manuscript with proper citation.

Introduction needs to be revised. The importance of insilico studies, drug repurposing, virtual screening techniques etc needs to be discussed with proper references. Following articles might be useful for this can include with proper citation.

<https://doi.org/10.1007/978-981-99-1316-9_11>
<https://doi.org/10.1201/9781003347705-5/>

The Introduction has been updated to underscore the critical role of in silico studies, drug repurposing, and virtual screening techniques in expediting drug discovery by efficiently identifying potential drug candidates early in development. These advancements are supported by relevant literature citations.

Addition of bioactivity prediction also provide important information for hypothesis of target mechanism predictions with proper citation. Following article may helpful.

<https://doi.org/10.3897/folmed.61.e47965>
The bioactivity prediction has been integrated, highlighting its value in hypothesizing target mechanisms.
Author should include limitations of computational studies for that following article may helpful and should be included with proper citation.

<https://doi.org/10.1016/B978-0-323-90608-1.00006-X>
<https://doi.org/10.1016/j.drudis.2020.07.005>

The manuscript now acknowledges the inherent limitations of computational studies, emphasizing challenges associated with relying solely on in silico methodologies.
The importance of virtual screening in drug discovery should be discussed with the examples of studies with proper citation for that that following article may helpful to author and should be included with proper citation.

<https://doi.org/10.1007/s42250-023-00611-9>
<https://doi.org/10.3390%2Fbiom11121877>
<https://doi.org/10.1007/s13205-023-03912-5>
<https://doi.org/10.1080/10799893.2020.1800734>

The importance of virtual screening in drug discovery has been thoroughly discussed, showcasing recent examples that demonstrate its efficacy in optimizing the identification of potential drug candidates.

Critical revision needed in discussion portion.

The Discussion section has been extensively revised to provide comprehensive insights into the significance of in silico techniques and the pivotal role of virtual screening in drug discovery, ensuring clarity and robust discussion of the findings and their implications.
